# Multicystic Intraosseous Ameloblastoma With Follicular Pattern in a 16-Year-Old Male: A Case Report

**DOI:** 10.7759/cureus.88103

**Published:** 2025-07-16

**Authors:** Benjamin Henderson, Jeung Woon Lee

**Affiliations:** 1 School of Dental Medicine, Lake Erie College of Osteopathic Medicine, Bradenton, USA

**Keywords:** braf v600e, follicular pattern, mandibular tumor, multicystic ameloblastoma, pediatric odontogenic tumor

## Abstract

Ameloblastoma is a benign, slow-growing, locally aggressive tumor derived from dental lamina and odontogenic epithelium. Though mainly occurring in adults, its presence has also been reported in the pediatric population. From the three subtypes of this neoplasm, the conventional (solid/multicystic) variant shows high rates of recurrence, possibly associated with the gain-of-function mutation in the BRAF V600E MAPK pathway.

A 16-year-old African American male presented with swelling, cortical expansion of the right mandible extending from the midline to the third molar region. Cone-beam computed tomography revealed an extensive multilocular radiolucency with a soap bubble or honeycombed appearance. Histopathological examination after incisional biopsy confirmed a multicystic intraosseous follicular pattern ameloblastoma. Presence of reverse polarity of basal cells and cystic structures lined by ameloblastic epithelium with wall invasion were observed.

This case highlights the presentation of conventional ameloblastoma in an adolescent. Conventional (solid/multicystic) ameloblastoma in this patient group requires careful consideration of treatment plan. Radical resection with adequate margins, often combined with immediate reconstruction like fibular free flap, offers the best chance for preventing potential for recurrence.

## Introduction

Ameloblastoma, as defined by the World Health Organization (WHO), is a benign and slow-growing tumor derived from the odontogenic epithelium of the dental lamina, enamel organ, rests of Malassez, and the basal cells of the oral mucosa. Although ameloblastoma rarely metastasizes, its clinical presentation displays aggressive infiltration into the surrounding bone and soft tissues, causing extensive local destruction with a high rate of recurrence when treated insufficiently [1]. Ameloblastoma constitutes the second most frequent odontogenic tumor, accounting for about 1% of tumors and cysts developing in the jaw [2,3].

Recent studies have reported a high rate of mutation of the BRAF gene (V600E substitution), in the mitogen-activated protein kinase (MAPK) pathway, as a critical event in the pathogenesis of ameloblastoma [4]. This gain-of-function mutation causes activation of the MAPK/ERK cascade, promoting uncontrolled cell proliferation, survival, leading to local tissue infiltration. Guimarães et al. (2021) and Kurppa et al. (2014) report that 63% to 82% of cases displayed BRAF V600E mutations [5,6]. In addition to the BRAF mutation, overexpression of NOTCH4 and the Sonic Hedgehog (SHH) signaling pathways, as well as the increased expression of the bone resorption regulator RANK/OPG, have also been reported in ameloblast cases [7-9].

Of the four main types of ameloblastoma, under the WHO classification, the most common subtype is the conventional (solid/multicystic), with the unicystic, peripheral (extraosseous), and metastasizing subtypes comprising the rest. Although conventional ameloblastoma is typically diagnosed in adults aged 30 to 60, recent studies also report its occurrence in patients under 14 to 18 years old at 10% to 25% [10,11]. Several studies that examined the Nigerian pediatric populations report a higher pediatric incidence and a predominance of the solid/multicystic type in this age group, whereas others suggest unicystic ameloblastoma being more common in Western children [12-14]. While conservative treatments like enucleation and curettage have been reported, they are associated with a high recurrence rate of 40% to 90%, leading in favor of a more aggressive radical resection, such as a marginal or segmental mandibulectomy [15,16].

Radiographically, the conventional type often exhibits a multilocular ("soap bubble" or "honeycomb") radiolucency with scalloped borders. Histologically, the epithelial islands with peripheral columnar cells show reverse polarity and central stellate reticulum-like areas.

Ameloblastoma among adolescents frequently manifests as a painless, progressive expansion of the jaw, with discernible facial asymmetry [11]. While asymptomatic at initial stages, larger lesions may induce dental mobility, malocclusion, or functional impairments of mastication and articulation. Characteristically, it consistently localizes to the posterior mandible, presenting radiographically as unilocular or multilocular radiolucencies [17]. Given the subtle early clinical indicators, adolescent ameloblastomas are commonly detected during routine dental imaging. Consequently, any persistent mandibular or maxillary enlargement, even in the absence of pain, necessitates prompt diagnostic evaluation to facilitate timely management.

We present a case report on a 16-year-old male patient diagnosed with a multicystic intraosseous ameloblastoma featuring a follicular pattern. This report aims to describe the clinical presentation, diagnostic journey, histopathological findings, and proposed management for this relatively rare adolescent case.

## Case presentation

**Figure 1:**
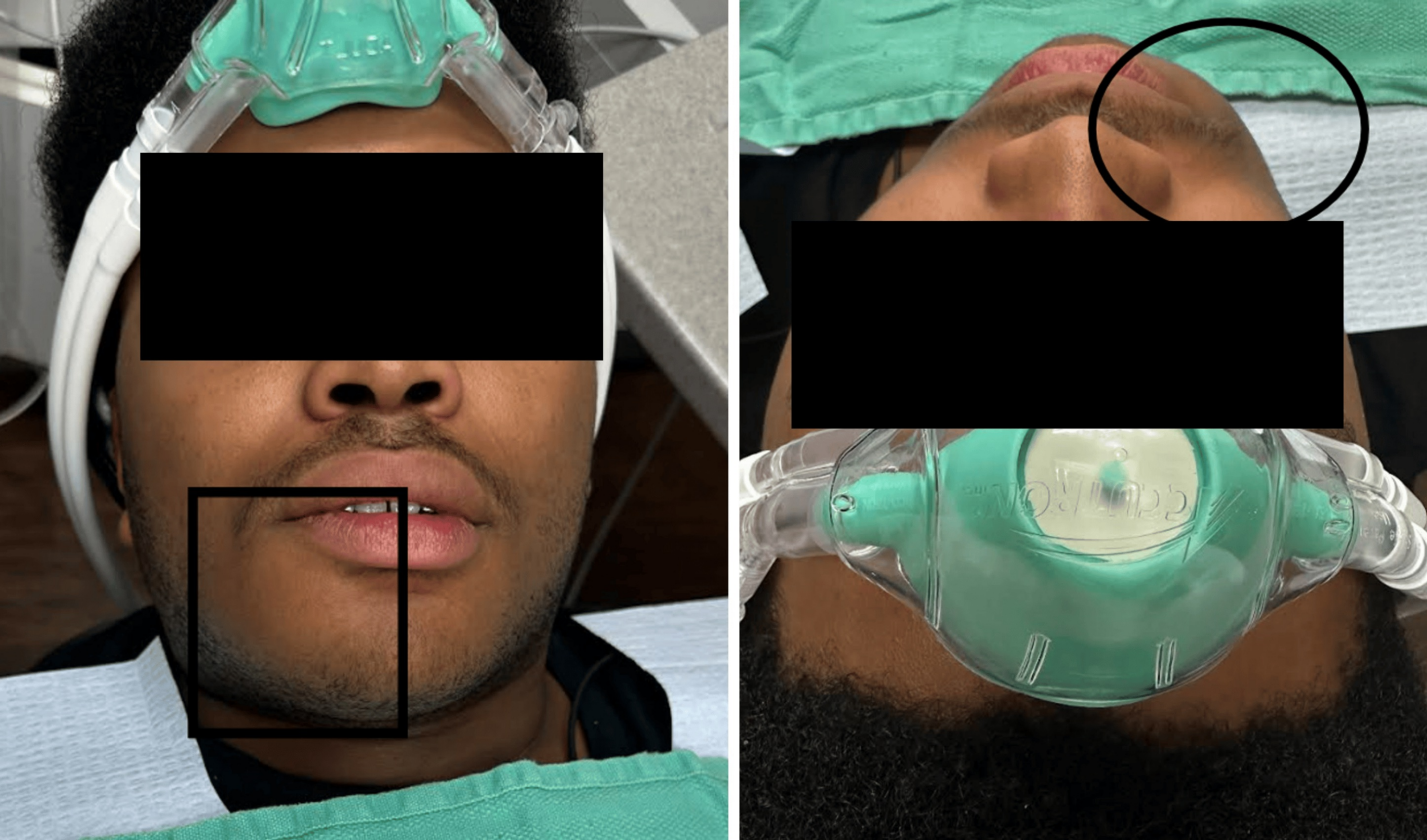
Anterior (A) and superior views (B) showing the expansion of the affected right jaw.

A 16-year-old African American male presented to the clinic with a chief complaint of swelling and cortical expansion in the right mandible. The swelling extended from the mandibular midline posteriorly to the third molar region. Extraoral examination revealed noticeable facial asymmetry due to this expansion (Figure 1).

The patient reported that this painless, progressive expansion had been present for six months. Initial clinical examination confirmed a firm, non-tender, expansile lesion involving the mid-body of the right mandible, extending into the buccal vestibule, with no overt signs of infection. Dental assessment revealed stable occlusion and absence of tooth mobility or obvious carious lesions. Panoramic imaging demonstrated a large multilocular radiolucency occupying the entire body of the right mandible, with associated root resorption of teeth #28 and #30, and displacement of tooth #28. The patient reported no associated paresthesia.

**Figure 2:**
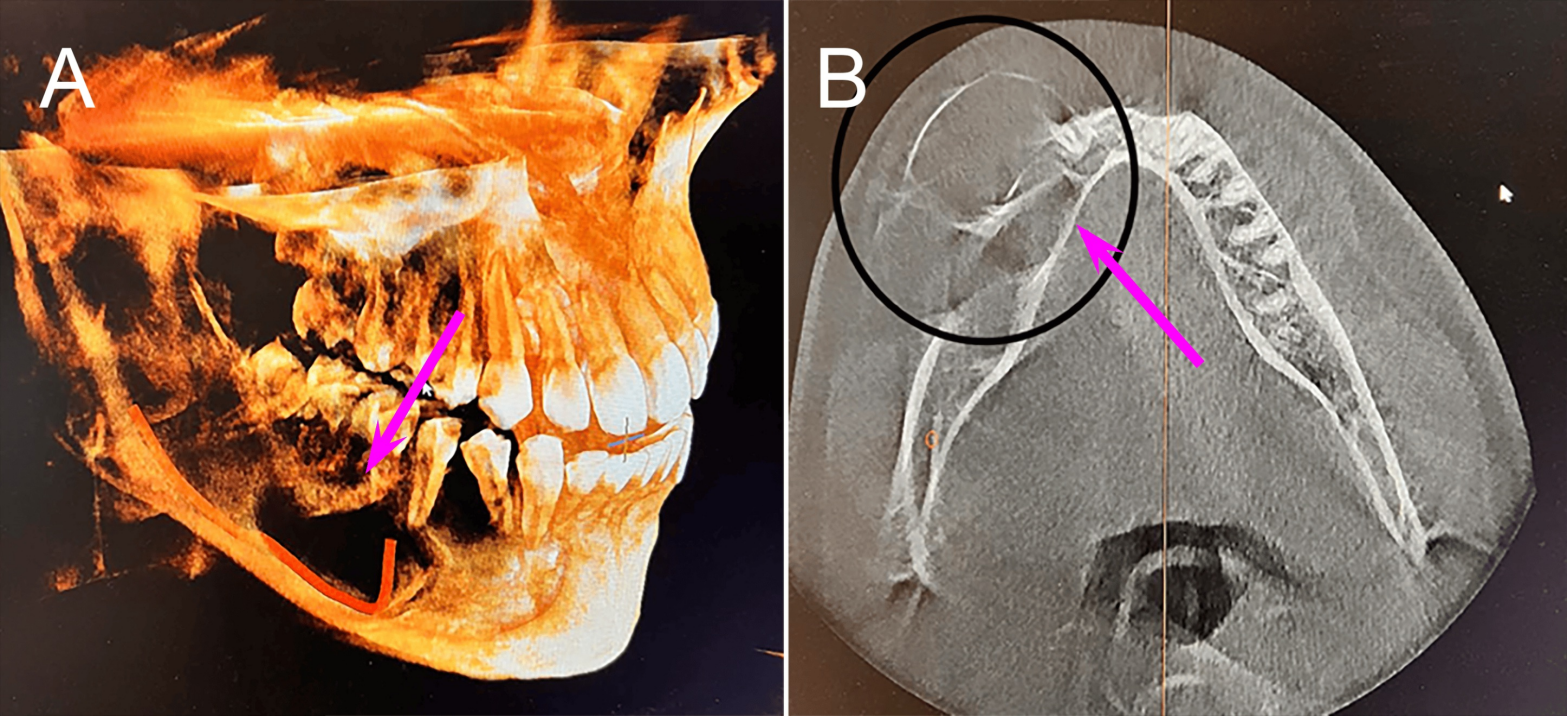
Cone-beam computed tomography (CBCT) radiographs of the right jaw showing the “soap-bubble” appearance. Lateral (A) and inferior views (B) of the affected right jaw.

Cone-beam computed tomography (CBCT) was performed, revealing an extensive, well-defined multilocular radiolucent lesion involving the right body and ramus of the mandible. The lesion exhibited the characteristic "soap bubble" appearance associated with ameloblastoma. Significant cortical expansion was also evident (Figure 2).

**Figure 3:**
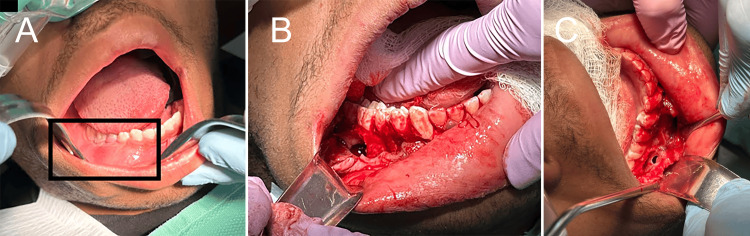
An incisional biopsy was followed by enucleation and curettage (ENC). Before (A) and after ENC (B, C) of the affected right jaw.

Intraoral examination confirmed bony expansion, particularly of the buccal cortical plate in the affected region (Figure 3).

An incisional biopsy with enucleation and curettage (ENC) of accessible lesional tissue was performed under anesthesia. The harvested tissue appeared as thin-walled, friable aggregates with brownish liquid secretions. No gross bone was identified in the tissue samples.

**Figure 4:**
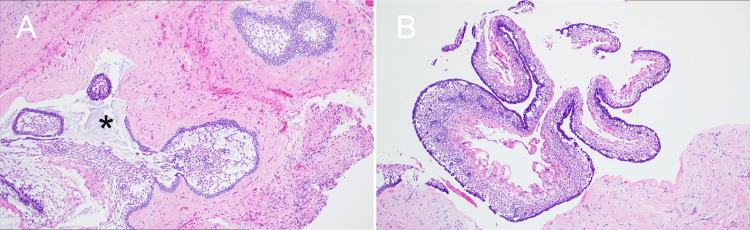
The paraffin blocked thin section stained with Hematoxylin and Eosin showed multicystic structures (A; 4x magnification). The peripheral follicular cells showed hyperchromatic nuclei with reverse polarity (B; 4x magnification).

Hematoxylin and eosin-staining of the incisional biopsy sections showed multiple cystic structures lined by odontogenic epithelium. Solid islands and follicles of neoplastic epithelial cells, resembling the enamel organ, were embedded within a mature fibrous connective tissue stroma. The peripheral cells of the follicles were columnar, exhibiting hyperchromatic nuclei and characteristic reverse polarity (nuclei polarized away from the basement membrane), consistent with ameloblast-like cells, sometimes described as having a “piano key” or "picket fence" arrangement. Central areas within the follicles contained loosely arranged, stellate reticulum-like cells. Areas of acanthomatous change (squamous metaplasia) within the epithelial islands were also observed, along with scattered mixed inflammation, fragments of bone, and hemorrhage. Based on these features, a final diagnosis of multicystic intraosseous ameloblastoma with a follicular pattern was established (Figure 4).

**Figure 5:**
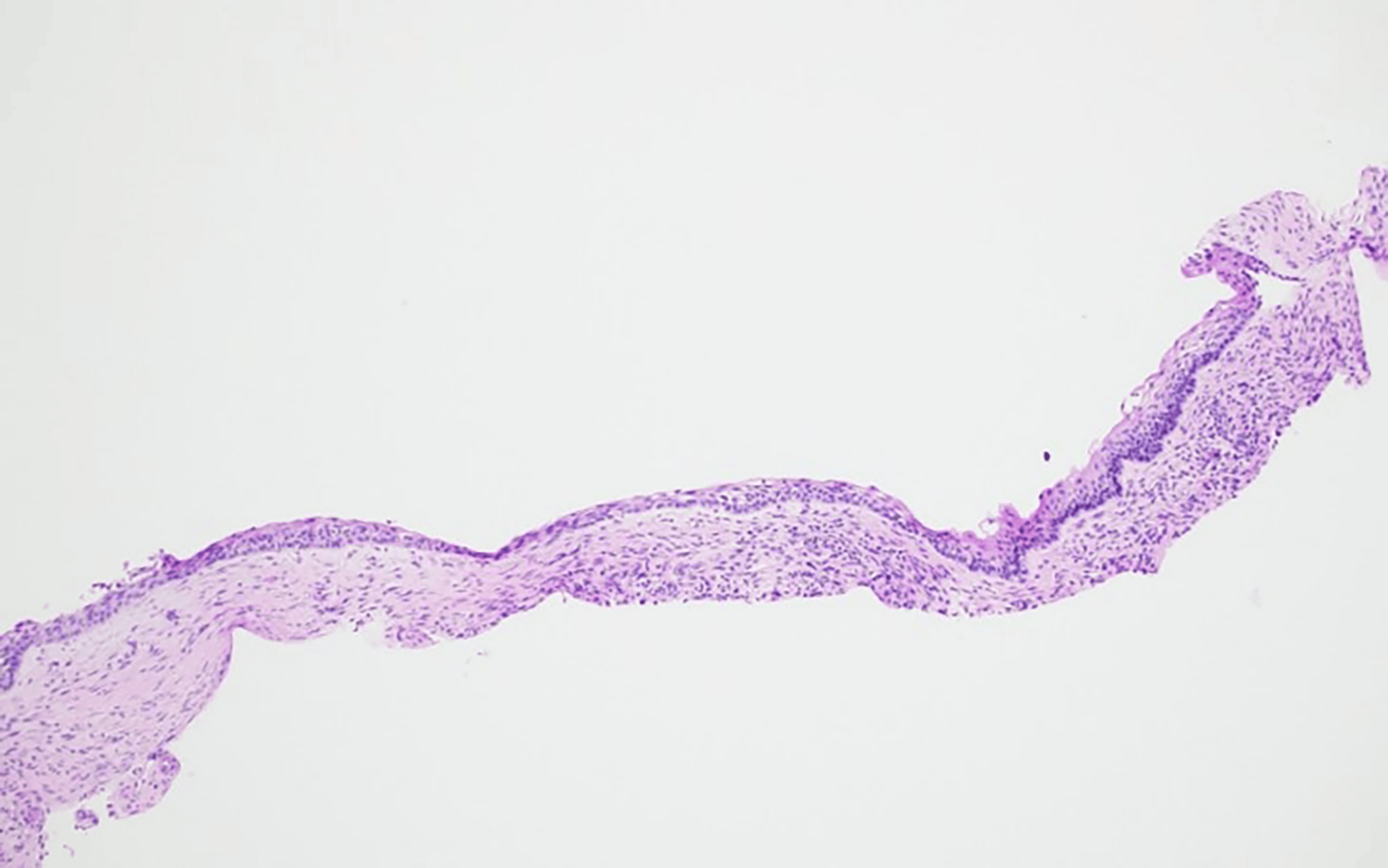
A section of radicular cyst showing peripheral palisading (Hematoxylin and Eosin staining; 4x magnification). There is a fence-like arrangement of the outermost edge of the tissue, near the connective tissue.

Evidence of tumor invasion into the cyst wall was noted (Figure 5).

## Discussion

Ameloblastoma in the pediatric and adolescent population is less frequent than in adults, accounting for approximately 10-15% of all cases, though its prevalence is notably higher in Africa and Asia (up to 25%) [18]. This case of a 16-year-old male falls within the typical age range reported for adolescent ameloblastoma with the mean age of 15 years. The mandibular location is also characteristic, as 80% to 95% of ameloblastomas arise in the posterior region of the mandible [19,20].

The diagnosis of conventional (multicystic) ameloblastoma with a follicular pattern was confirmed through the histopathological analysis. The presence of epithelial islands resembling the enamel organ, peripheral columnar cells with reverse polarity and hyperchromatic nuclei, and central areas mimicking stellate reticulum indicated the lesion as an ameloblastoma. Additionally, several large clinical studies of Nigerian children have found the solid/multicystic variant to be predominant even in those under 20 years of age [12,21]. The presence of invasive characteristics in the cyst wall further supported the classification.

A critical implementation in managing conventional ameloblastoma is complete surgical removal with adequate margins to prevent recurrence. Conservative treatments using enucleation and curettage are associated with very high recurrence rates for multicystic variant at 50% to 90%. In contrast, radical resection achieves significantly better local control, with recurrence rates of 8% to 20% [15,22]. While more conservative approaches might be considered for some unicystic subtypes or very young children to mitigate impact on growth, the multicystic nature and invasiveness in this adolescent patient strongly favors radical resection.

The extensive mandibular defect resulting from resection necessitates reconstruction to restore function (mastication, speech) and facial aesthetics. The vascularized fibula free flap procedure, compared to multistage conservative surgical technique (dredging method), reported high success rates between 72% to 82% with a reduction in 14-item Oral Health Impact Profile (OHIP-14) score from 12.0 to 8.3 [23]. Its advantages, particularly relevant in pediatric and adolescent patients, include providing a long segment of vascularized cortical bone, reliable blood supply via peroneal vessels, potential for osseointegration allowing subsequent dental implant placement, low donor site morbidity, and the ability to incorporate a soft tissue paddle for intraoral lining or external coverage. Immediate reconstruction generally leads to better functional and aesthetic outcomes compared to delayed procedures. Preserving the temporomandibular joint, when oncologically safe, is also crucial for maintaining mandibular function.

Long-term follow-up is imperative after treatment for ameloblastoma, regardless of the initial approach, due to the possibility of late recurrence. Regular clinical and radiographic surveillance may allow for early detection and management of any recurrent disease. The identification of the BRAF V600E mutation may offer further possibilities for targeted usage of BRF inhibitor therapies, especially in recurrent or unresectable cases [24]. Surgery, however, remains the primary modality.

Differential diagnosis for adolescent ameloblastoma necessitates careful consideration of other jaw pathologies with similar clinical and radiographic presentations. The odontogenic keratocyst often mimics unicystic ameloblastoma, presenting in the posterior mandible as unilocular or multilocular radiolucencies with minimal osseous expansion, exhibiting shared features such as jaw swelling and dental displacement [25]. Similarly, the dentigerous cyst, a common adolescent presentation associated with unerupted teeth, manifests as a well-defined unilocular radiolucency that primarily displaces dentition, necessitating differentiation from the aggressive osseous destruction characteristic of ameloblastoma [25]. Additionally, central giant cell granuloma, prevalent in patients under 30 years of age, frequently involves the anterior mandible, may cross the midline, and exhibits both unilocular and multilocular radiographic features with significant osseous expansion and cortical thinning [26]. Ultimately, precise diagnosis depends upon the careful integration of comprehensive clinical findings, advanced radiographic imaging, and definitive histopathologic analysis to guide optimal patient management.

## Conclusions

Conventional (multicystic) ameloblastoma, typically presenting in adults, can affect adolescents, requiring management tailored to both oncologic control and considerations for facial growth. This case of an extensive follicular ameloblastoma in a 16-year-old male underscores the importance of accurate histopathological diagnosis and imaging to guide treatment. Radical en bloc resection with appropriate margins remains the treatment of choice for conventional ameloblastoma to minimize the high risk of recurrence. Immediate reconstruction with vascularized bone flaps (fibula free flap) would provide excellent functional and aesthetic outcomes for younger patients.
